# Non-spherical Polymeric Nanocarriers for Therapeutics: The Effect of Shape on Biological Systems and Drug Delivery Properties

**DOI:** 10.3390/pharmaceutics15010032

**Published:** 2022-12-22

**Authors:** Prescillia Lagarrigue, Filippo Moncalvo, Francesco Cellesi

**Affiliations:** Dipartimento di Chimica, Materiali ed Ingegneria Chimica “G. Natta”, Politecnico di Milano, Via Mancinelli 7, 20131 Milan, Italy

**Keywords:** polymeric nanocarriers, non-spherical, drug delivery, filomicelles, nanoworms, nanorods, nanodisks

## Abstract

This review aims to highlight the importance of particle shape in the design of polymeric nanocarriers for drug delivery systems, along with their size, surface chemistry, density, and rigidity. Current manufacturing methods used to obtain non-spherical polymeric nanocarriers such as filomicelles or nanoworms, nanorods and nanodisks, are firstly described. Then, their interactions with biological barriers are presented, including how shape affects nanoparticle clearance, their biodistribution and targeting. Finally, their drug delivery properties and their therapeutic efficacy, both in vitro and in vivo, are discussed and compared with the characteristics of their spherical counterparts.

## 1. Introduction

Polymeric nanoparticles have been extensively studied in the last decades as therapeutics nanocarriers, especially for personalized medicine applications. They can be designed to target tissues and to overcome biological barriers which are specific to disease states and the patient subset; thus, an individualized treatment plan can be developed, minimizing the impact of patient heterogeneity and improving drug specificity [1,2]. Drug encapsulation in such nanoparticles allows therapeutics efficiency to be increased and side effects decreased at the same time. The high degradability and low molecular weight of the drug often lead to rapid clearance and a short circulation half-life after injection [3,4]. Polymeric nanocarriers allow these drawbacks to be overcome thanks to their biocompatibility, tunable biodegradability, capacity to overcome biological barriers and their targeting abilities by modification of their surface. In addition to these properties, the design of nanoparticles was shown to have an important impact on their behavior both on biological process and on targeting drug delivery properties. So far, several studies have investigated the impact of the size of spherical polymeric carriers on their behavior in vitro and in vivo, and on drug delivery properties [5,6,7,8]. Although the nanoscale size of the carriers has been demonstrated to be an important parameter to improve drug delivery and therapeutics efficiency, the shape of the nanoparticle also plays an important role in different biological processes [9]. Anisotropic nanocarriers present different interaction with both drugs and cells, and their higher surface area leads to a higher capacity of drug encapsulation and delivery, due to localized degradation, enhanced targeting through a higher surface area for cell binding, and easier interaction with targeted cells [10,11,12,13].

This review aims to describe the importance of polymeric nanoparticle shape on their properties as therapeutics nanocarriers. Firstly, the main manufacturing methods are described: self-assembly process, membrane stretching and particle replication in nonwetting templates. Then, the interactions of such obtained anisotropic nanocarriers with biological barriers are presented. Finally, their properties as drug delivery systems, both in vitro and in vivo, are discussed.

## 2. Fabrication of Non-spherical Polymeric Nanoparticles

**Figure 1:**
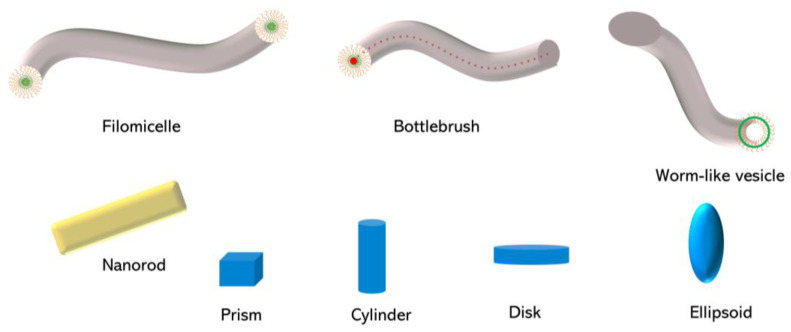
Most common non-spherical polymeric nanoparticles used in drug delivery.

Various methods described in the literature [2,12,13,14] can be used to produce non-spherical polymeric nanoparticles for drug delivery applications (Figure 1). This review mainly focuses on the most common methods of bottom-up and top-down approaches, summarized in Table 1, and describes them succinctly.

### 2.1. Self-Assembly Techniques

**Figure 2:**
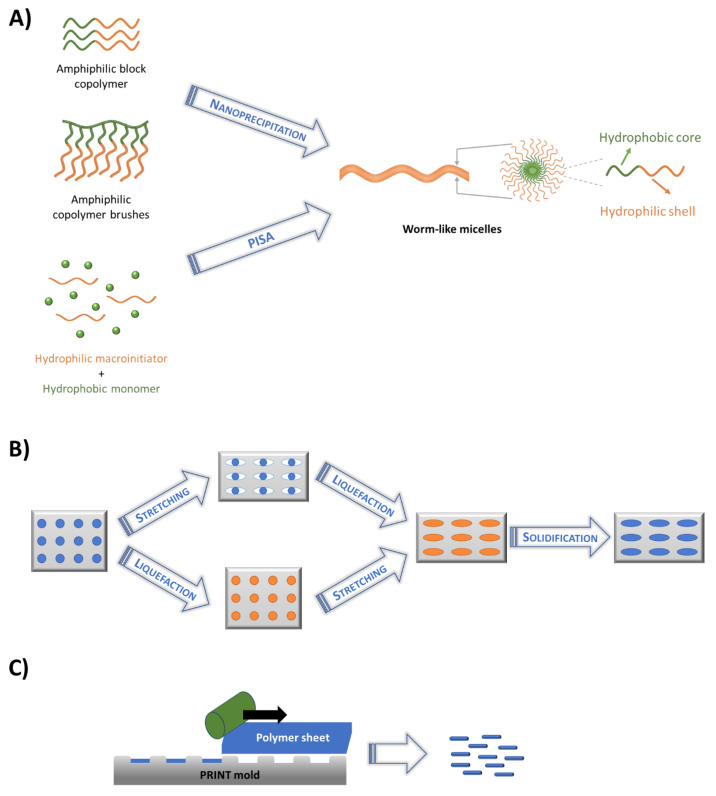
Presentation of the main processes to manufacture non-spherical nanocarriers: (**A**) Self-assembly techniques (orange: hydrophilic group, green: hydrophobic group), (**B**) Membrane stretching method. The film containing the initial nanoparticles is stretched before or after the liquefaction step, which is obtained either by particle solubilization in adequate solvent or by heating above the glass transition temperature, (**C**) Particle replication in nonwetting template (PRINT). This soft lithography technique utilizes highly fluorinated nonwetting molding template to create nano-scale patterns.

The typical bottom-up method to produce non-spherical nanoparticles is based on self-assembly of amphiphilic block copolymers [4]. This process is based on thermodynamic equilibrium, intermolecular interactions and chain packing driven by the different polarities of the copolymer blocks. The amphiphilic composition permits the encapsulation of lipophilic drugs in the hydrophobic core, which is protected by the hydrophilic shell. Different structures, such as spherical, cylindrical, or filomicelles (worm-like micelles), can be obtained [4,42,43,44]. Particle shape and size depends on the polymer/aqueous phase separation mechanism, which is influenced by different factors such as polymer composition (monomeric units, architecture, molecular weight of each block) and self-assembly conditions (organic solvent, water addition, stirring, evaporation, temperature). Two main self-assembly procedures can be highlighted: the conventional self-assembly process leading to micelle formation, and the polymerization-induced self-assembly (PISA), as presented in Figure 2A.

The **conventional self-assembly procedure** is based on two main steps: (1) the block copolymers synthesis and (2) the formation of the final nanoparticles. Once the amphiphilic block copolymers are synthesized, the nanoparticles are manufactured by nanoprecipitation. The amphiphilic block copolymers are dissolved in a good solvent for all blocks and then added dropwise to a solvent in which the hydrophilic group is soluble. Alternatively, the polar solvent is slowly added to the polymer solution. The difference of solvent polarity leads to the assembly of micelles, with the hydrophobic groups forming the particle core and the hydrophilic ones the corona. Finally, the volatile organic solvent is evaporated [4,45].

Linear diblock copolymers are the most studied. In 1995, Zhang et al. were the first to described non-spherical nanoparticles synthesized by this method using polystyrene-b-poly(acrylic acid) copolymers (PS-b-PAA) [20]. They demonstrated that different shapes can be obtained by tuning the hydrophilic block degree of polymerization and thus its molecular weight. They obtained spherical micelles using PS_200_-b-PAA_21,_ as well as worm-like micelles by decreasing the degree of polymerization of PAA (using PS_200_-b-PAA_15_). Numerous investigations were then carried out to obtain non-spherical nanoparticles by tuning either the diblock copolymers or the self-assembly parameters. As a few examples, rod-like nanoparticles were obtained with polystyrene-b-poly(ethylene oxide) PS_240_-b-PEO_80_ [21], and filomicelles with nanometric diameters (22–60 nm) and different length (1-18 µm) using polyethylethylene-b-poly(ethylene glycol) (PEE_35_-b-PEG_42_), polycaprolactone-b-poly(ethylene glycol) (PCL_24_-b-PEG_44_ and PCL_58_-b-PEG_110_) [15], poly(ethylene glycol)-b-poly(propylene sulfide) (PEG_45_-b-PPS_44_) [22], series of poly(ethylene glycol)-polylactide (PEG-b-PLA) diblock copolymers [23]. By tuning the solution conditions, PS_310_-b-PAA_52_ block copolymers can lead to worm-like micelles or vesicles [14].

Ma and coworkers also demonstrated that triblock copolymers based on poly(acrylic acid)-b-poly(methyl acrylate)-b-polystyrene (PAA_90_-b-PMA_80_-b-PS_100_) formed rod-like micelles in presence of water soluble carbodiimide [18]. Although self-assembly of diblock and triblock copolymers may allow different nanoparticle shapes to be obtained for drug delivery applications, the resulting nanoparticles often present poor colloidal stability and therefore poor drug delivery properties. To overcome these limitations, some investigations were performed on non-spherical cross-linkable micelles, obtained by addition of cross-linking agents which can react with one or both functional blocks [24,44]. Yang et al. demonstrated that crosslinked worm-like vesicles based on triblock copolymers of PEG and poly(lactic acid) (PEG-PLA-PEG) presented higher stability in vivo [19]. Non-spherical morphology was also obtained by complexation of block copolymers with nucleic acids for gene delivery. Spherical, rod-like and worm-like DNA-based nanoparticles were synthesized from PEG-polyphosphoramidate (PPA) block copolymer [46], linear PEI (lPEI)-g-PEG copolymers [25,26], as well as the thermo-responsive ABC triblock copolymers consisting of poly(2-ethyl-2-oxazoline) (PEtOx), poly(2-n-propyl-2-oxazoline) (PnPrOx) and poly(l-lysine) [47].

Amphiphilic polymer brushes are another interesting alternative to prepare non-spherical nanocarriers in order to improve their stability in blood circulation and their drug release profile [48,49]. Müllner et al. performed unimolecular nanoworm micelles based on branched amphiphilic copolymers of polycaprolactone-b-(poly[(ethylene glycol) methyl ether methacrylate]-co-glycidyl methacrylate) (PCL-b-(PEGMA-co-GMA)) and showed that the aspect ratio of such nanoworms affected the in vivo circulation time [28]. High-molecular weight poly(l-glutamic acid) based brush polymers, were synthesized by a combination of ring-opening metathesis polymerization of norbornene-based monomers and ring-opening polymerization of γ-benzyl-l-glutamate n-carboxyanhydride [31]. These brush polymers were conjugated with a model drug, camptothecin (CPT), to obtain unimolecular nanocarriers, which were characterized in terms of stability, drug release kinetics, and in vitro toxicity. Li and coworkers showed that the same triblock amphiphilic copolymer of polystyrene-b-poly(methyl acrylate)-b-poly(tert-butyl acrylate) formed spherical micelles in a linear conformation and led to cylindrical micelles in a brush conformation [27].

**Polymerization-induced self-assembly** (PISA) is an innovative micelles self-assembly technique (Figure 2A). During the last decade, this process has been widely studied, leading to the synthesis of nanocarriers for drug delivery with different shapes such as spheres, worms, rods, vesicles which can be stimuli-responsive [50,51]. This procedure is based on an amphiphilic block copolymers synthesis by reversible-deactivation polymerization, followed by an in-situ non-spherical micelles formation. A hydrophilic macroinitiator is firstly synthesized, and then dissolved in a selected solvent to perform chain extension in the presence of the second monomer, either by dispersion or emulsion. The second block becomes insoluble while its degree of polymerization increases, leading to self-assembly formation of differently shaped nanoparticles. For instance, worm-like rods or vesicles nanoparticles of poly[oligo(ethylene glycol) methacrylate]-b-[poly(styrene)-co-poly(vinyl benzaldehyde)] (POEGMA-b-P(ST-co-VBA)) were obtained by changing the degree of polymerization of ST-co-VBA block, which permitted a controlled release of doxorubicin (DOX) [32]. Different photosensitive non-spherical nanocarriers based on Poly(N-(2-hydroxypropyl)methacrylamide)-b-poly(2-nitrobenzyl methacrylate-co-7-(2-Hydroxyethoxy)-4-methylcoumarin PHPMA-b-(NBMA-co-CMA), and obtained through PISA procedure also showed interesting performances for DOX encapsulation and delivery [33]. Very recently, a ‘living’ crystallization-driven self-assembly (CDSA) was proposed for the preparation in water of rod-like polymer nanoparticles based on poly(2-oxazoline)s (POx) [34]. The resulting in POx nanorods (length between 60 and 635 nm) exhibited stealth behavior and excellent biocompatibility both in vitro and in vivo, with low immune cell association and relatively high blood circulation time.

Even though self-assembly is an efficient method to synthesize non-spherical nanoparticles, the control of shape and size of the nanocarriers may be limited by the polymerization conditions and self-assembly mechanisms. To overcome these drawbacks, top-down approaches has emerged in the last years, to manufacture nanoparticles of controlled shape and size, with interesting scalability. Among these different techniques, membrane stretching, lithography and molding techniques are the most promising. 

### 2.2. Membrane Stretching Technique

Membrane stretching is the most used method after self-assembly (Figure 2B) [4]. It is based on the mechanical deformation of pre-manufactured spherical nanoparticles into complex shapes. Indeed, the nanoparticles geometry is determined post-synthesis, by casting into poly(vinyl alcohol) membranes, liquefying and stretching to reach anisotropic shapes. Two alternative methods can be used, as the film containing the nanoparticles can be stretched either before or after the liquefaction step. The latter can be reached either by solubilization of the polymer nanoparticles in adequate solvent or by heating them over the polymer glass transition temperature [44,52]. Through this method, anisotropic nanocarriers such as rods, elliptical disks and barrels were obtained for antibody immobilization and display; however a limited number of polymeric materials can be used, such as polystyrene (PS) and poly(lactic-acid-co-glycolide) (PLGA) [35,36,37].

### 2.3. Particle Replication in Nonwetting Template (PRINT)

Among the different top-down techniques, lithography and molding methods are widely used to control polymeric nanoparticles shape. Photolithography techniques allow polymer nanoparticle manufacturing with a resolution of 10 nm [53]. Different geometries are available to tailor the molds. The most famous and used technique is the particle replication in nonwetting template (PRINT) method, a soft lithography technique that utilizes highly fluorinated nonwetting molding template to create nano-scale patterns (Figure 2C). Discrete and well-defined nanoparticles with cubic, cylindrical, cone, rod and worm-like shapes were obtained without any residual film coming from the interface between polymer solution and fluorinated mold, which is one of the main drawbacks of classic lithography techniques [40]. Nanoparticles were typically formed from PEG, PLA and PLGA [4,41,44] and exploited as nanocarriers for the delivery of chemotherapeutic drugs and proteins [38,39,40].

## 3. Effect of Particle Shape on Overcoming Biological Barriers

**Figure 3:**
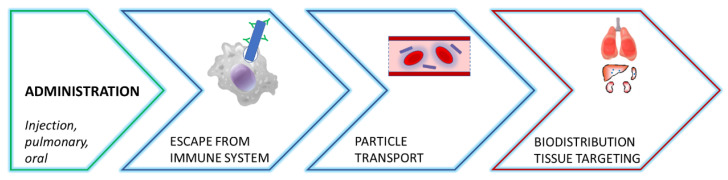
Different steps from nanocarriers administration to tissue targeting.

Even if the interactions between spherical nanoparticles and biological systems have been widely studied, the manufacturing advancement to control particle shapes opened new opportunities to overcome biological barriers [54]. While various administration routes have been explored for spherical nanoparticles, including transdermal, oral, and ocular administration, most of the non-spherical polymeric nanocarriers are administered using intravenous or inhalation procedures [55]. Recent studies have highlighted the importance of the geometry of nanovector in different biological processes, from their injection/inhalation to their internalization by the targeted cells (Figure 3), namely interaction with immune system, nanoparticles transport and their biodistribution and targeting behavior. The main effects of nanocarriers shape on these different biological processes are summarized in Table 2.

### 3.1. Interactions with Immune System

Regarding the interactions with the immune system, phagocytosis by macrophages is the first biological process that nanocarriers must overcome after their administration. Investigations on biological behavior of non-spherical nanoparticles have highlighted the crucial role played by nanocarriers geometry in their uptake by immune cells [56,71,83,84]. This two-steps mechanism is based on the adhesion of nanoparticles to the surface of immune cells (macrophages, monocytes, neutrophils etc.) followed by their internalization. 

As it occurs in nature with cylindrical bacteria such as *E. coli* [85], several studies demonstrated that elongated nanoparticles such as worms [28,56,57,58,60,86] or ellipsoids [56,70] are able to reduce or inhibit phagocytosis rate and thereby evade the immune response. More specifically, Champion et al. investigated in detail the phagocytosis mechanism on polystyrene nanoparticles of different shapes, including spheres, ellipsoids, elliptical or rectangular disks and UFOs [56]. The results demonstrated that phagocytosis can be initiated for any type of shape in at least one orientation. However, they underlined the importance of local particle shape at the point of cell attachment during the first step of the phagocytic mechanism. In fact, the internalization and its velocity are inversely correlated with tangent angles (Ω) formed between cells and nanocarriers. Briefly, particles are internalized by macrophages through acting-cup and ring formation when Ω < 45°, while in the other case, cells spread on the nanoparticles but are unable to internalize them. Hence, the curvature of particles has a determinant role in their internalization independently of their size. In addition, other investigations showed that a high aspect ratio led to a decreased internalization, as demonstrated for worm-like polymeric nanoparticles with an aspect ratio higher than 20 [57,58]. Nanocarrier flexibility also affect cellular uptake, as stiffer particles typically showed increased internalization by immune cells [87]. Flexible filomicelles based on tetrablock copolymers of PEG-b-PPS linked by a pi-stacking perylene bisimide (PBI) [72], were optimized to decrease macrophage uptake and increase circulation time after intravenous administration in mice. 

Moreover, numerous works highlighted that the presence of hydrophilic polymer at the surface of nanocarriers, such as PEG, may decrease the macrophage clearance rate by reducing opsonization [88]. Müllner et al. synthesized two types of anisotropic nanoparticles based on PEGMA-co-PGMA or PCL-b-PEGMA-co-PGMA cylindrical polymer brushes [28]. Their work demonstrated that particle clearance is three times higher for long PEGMA-co-PGMA brushes (up to 1000 nm) in comparison to the smallest spheres (35 nm). Moreover, a comparison between the longest PEGMA-co-PGMA and PCL-b-PEGMA-co-PGMA cylindrical polymer brushes showed a more rapid clearance of the latter, which possessed a crystalline core. Mathaes et al. also demonstrated that the association of elongated shape and presence of PEG shell permit the strong reduction recognition and phagocytosis of PLGA nanoparticles [60]. 

In some cases, nanocarriers are designed to interact with immune cells and inhibit their activation, instead of avoiding immune recognition. PEG-b-PPS filomicelles were tested to deliver chloroquine to plasmacytoid dendritic cells (PDC) via passive, morphology-based targeting, in order to inhibit the production of type I interferon [22]. Cellular uptake and biodistribution studies showed a preferential accumulation in human PDC and monocytes in vitro and in tissues frequently damaged in systemic lupus erythematosus patients. 

These results highlight the importance of shape and size of the nanocarriers to decrease phagocytosis effect, although surface chemistry, density and rigidity also play a key role in clearance mechanism.

### 3.2. Particle Transport

Once the fast uptake by immune cells is avoided, the nanocarriers efficiency also depends on their transport from the administration site to the target organs. For nanocarriers injected intravenously, their transport characteristics are strongly influenced by margination phenomenon and their blood circulation half-life.

Margination phenomenon describes the capacity of nanosystems to escape from blood flow to vessel walls and diffuse inside the target organs. As opposed to spherical nanoparticles, which tend to stay between red blood cells flow and vessel walls, thus limiting their ability to margination, the non-spherical nanoparticles possess a more complex flow behavior [3,89]. Their aspect ratio directly influences their lateral drift velocity, enhancing their rotation and oscillation between walls. Moreover, non-spherical particles also present a higher specific surface area, leading to stronger adherence to the walls and therefore a higher binding possibility, as demonstrated for nanorods or nanodisks, in comparison to their spherical counterparts [68,69]. 

The circulation half-life of the nanocarriers is highly influenced by their shape, since geometry has a significant impact on phagocytosis and flow behavior as mentioned before. The reduced clearance and margination phenomena of non-spherical nanoparticles seems to extend circulation half-life when compared to the spherical ones. This trend was demonstrated in several studies, mainly on polymeric filomicelles and nanoworms [15,16,28,29,30,61,66], but also on nanorods [17] and nanodisks [79,81], and this shape effect was confirmed by similar tests on inorganic (silica, gold, iron oxide) nanomaterials [90,91,92].

Geng et al. demonstrated that self-assembled filomicelles from block copolymers had a circulation half-life 10-fold longer than spherical micelles of equivalent chemistry, and were present in rodent blood vessels for one week [15]. Moreover, they compared the behavior of filomicelles possessing different lengths (2 μm, 4 μm, 8 μm,18 μm). Results highlighted that the circulation half-life increased with the filomicelle length up to 8 µm (which is equivalent to the size of red blood cells), however the longest filomicelles (18 µm) presented the same behavior than the 8 µm ones. This may be related to a rapid fragmentation of long conventional self-assembled nanoparticles (>10 µm) which seemed to be less stable in blood flow, due to a combination of cell-interactions and shear forces, leading to fragments with a length under 10 µm after few days. Similar studies on nanorods [17] and nanodisks [79,81] based on amphiphilic block copolymers showed higher circulation half-lives in comparison to their spherical counterparts.

Müllner et al. investigated the effect of shape and rigidity of polymer brushes nanoworms on circulation half-life [28]. Interestingly, changes in rigidity had limited effect in vitro, although the incorporation of a crystalline core compartment into the brushes resulted in a more rapid clearance in vivo. This suggested that nanoworms flexibility may play a key role in their filtration and clearance. Despite the high molecular weights, these nanoworms presented high circulation half-life (over 20 h). However, in contrast to the results of Geng et al. for self-assembled filomicelles, the circulation half-life decreased with the increase of the nanoworm length. This trend was confirmed by the studies of Zhang et al. [29,30], and may be explained by the difference in stability between self-assembled materials and polymer brushes backbone. Concerning the shape effect, spherical polymer brush nanoparticles presented higher in vivo circulation half-life (≃6.2 h) than that of nanoworms with the same volume and surface chemistry (≃4.6 h) [29]. 

Other studies also investigated the transport abilities of non-spherical nanoparticles through the gastrointestinal barrier after oral administration. Particularly, Li et al. [69] and Banerjee et al. [78] showed that polystyrene nanorods obtained by membrane stretching possessed higher retention time and transport abilities through intestinal cells than their spherical counterparts. 

### 3.3. Biodistribution and Targeted Delivery

The shape of nanocarriers represents a key parameter that influences both their biodistribution and cell/tissue targeting in vivo. Besides clearance and transport, geometry also has a major role in the internalization by targeted cells and on the amount of targeting moieties that nanocarriers can display at their surface. Hence, in vivo studies have demonstrated that nanoparticle geometry impacts their penetration from vessels to tissue and their capacity of binding to the targeted tissues, by comparing filomicelles or nanoworms [16,28,29,30,61,64,65], nanorods [17,35,36,67,78] and nanodisks [35,69,81,93] to their spherical equivalents. 

Polymeric filomicelles represent a promising drug delivery system for targeting endothelial cells in the lumen of blood vessels, since they may adhere strongly, lengthwise to specific targeted moiety on cell surface. In fact, antibody-decorated filomicelles based on poly(ethylethylene)-b-poly(ethyleneoxide) diblock copolymer, which recognize distinct endothelial surface molecules, adhered to endothelium with high specificity both in vitro and in vivo [82]. Christian and coworkers investigated the biological behavior of PEO-b-CL filomicelles for paclitaxel (PTX) delivery in comparison to equivalent spherical micelles [61]. The results of the in vivo study on mice highlighted that filomicelles permit a higher tumor selectivity and reduce accumulation in off-target organs (heart, lung, liver, spleen) in comparison to their spherical counterparts. In addition to the shape-effect, Ke et al. have demonstrated that the length of the filomicelles also plays a role in their biodistribution and targeting capacity [16]. Comparing filomicelles of two different lengths (180 nm and 2.5 µm) with spherical micelles with the same diameter and composition, they demonstrated that short filomicelles are more efficient to target tumors than the long ones and spherical micelles. The formers are easily internalized due to their higher specific surface area. The presence of long filomicelles in tumor was limited as they mainly accumulated in liver, spleen, and lung. Similar results were found for brush-based nanoworms, as the shortest nanocarriers accumulated easier in tumors than spherical or long counterparts [29,30]. Moreover, the longer nanoworms presented high accumulation in spleen and liver.

Li et al. studied self-assembled PEG-PCL nanorods of different size loaded with DOX and demonstrated similar results in vivo. The accumulation of DOX in tumor was higher for short nanorods than for spherical or long counterparts, indicating a higher internalization of nanorods by the tumor cells. Moreover, it was noticed that liver and spleen accumulation were higher for sphere and long nanorod carriers [17]. The same trend was shown for PS nanorods and nanodisks coated with specific antibodies, manufactured by membrane stretching [35,36,69,78,81]. Specially, Kolhar and coworkers highlighted an increased endothelial specificity and brain accumulation of coated nanorods in comparison to their spherical equivalent [36], which presented a higher accumulation in lung and brain [36]. Same conclusions were also obtained by Muro et al. for PS antibody-coated nanodisks, which presented greater targeting specificity in mice lungs and lower liver uptake compare to nanospheres [81]. Barua et al. confirmed these results by studying the shape effect of antibody-coated PS nanoparticles on specific uptake and binding [35]. Nanorods presented the highest uptake and surface binding to breast cancer cells in vitro, and although nanodisks possessed lower specific uptake than nanorods, their capacity to enter and bind targeted cells was greater than that of spherical counterparts. In oral administration, the geometry seemed to play a similar role in targeted cell uptake. In fact, both nanorods and nanodisks presented higher uptake in intestinal cells than the spherical equivalents and a higher retention time [69,78].

Regarding lung vascular targeting via inhalation delivery, it has been reported that nanoparticles can also be internalized via caveolae-mediated endocytosis, with eventual translocation across endothelial cells of the lung vasculature. As filamentous influenza viruses induce transcytosis from alveolar space into the lung vascular space, filomicelles may also be transported through paracellular transport with a similar mechanism [63,94].

The role of nanoparticle aspect ratio in biodistribution and tumor penetration was also investigated with rod-shaped nucleoprotein nanoparticles with predetermined aspect ratios and surface decoration, either with PEG or receptor-targeted RGD [95]. PEGylated nanorods with the lowest aspect ratio achieved the most efficient passive tumor targeting due to their fast diffusion, whereas RGD-labeled particles with a medium aspect ratio achieved even more targeting efficacy because of the effect of ligand–receptor interactions. Since iRGD peptide is known to significantly improve the tumor accumulation and tumor penetration, cylindrical polymer brushes with a different iRGD conjugation density were evaluated in terms of cellular uptake, tumor targeting and permeability [77]. It was demonstrated that the highest conjugation density enhanced cellular uptake five times compared with iRGD-free brushes and tumor accumulation twice in subcutaneous 4T1 mammary tumor-bearing mice. These nanomaterials also provided a penetration depth in three-dimensional multicellular spheroids larger than 100 μm. 

Pharmacokinetic studies on nanotubes generated from cyclic peptide–poly(HPMA) conjugates showed that the large size of these particles reduced renal clearance and enhanced systemic circulation [62]. Their ability to slowly disassemble makes these materials promising for reducing organ accumulation during systemic drug delivery. 

Brush nanoparticles of different topologies were obtained from poly(2-(2-bromoisobutyryloxy) ethyl methacrylate) (PBIEM) at different degree of polymerization (DP), and side chains of PEGMA and glycidyl methacrylate (GMA) [74]. In vitro tests showed that nanorod shapes exhibit higher association and penetration into multicellular tumor spheroids compared to their spherical or filamentous counterparts.

Nanospheres or nanorods of different diameters and lengths were also obtained by varying the number of the conjugated hydrophobic drug camptothecin (CPT) in the amphiphilic PEG-block-dendritic polylysine–CPT (PEG–xCPT) copolymer [75]. Size and shape were found to strongly affect blood clearance, biodistribution and tumor targeting. The nanorods with medium lengths (<500 nm) had a much longer blood circulation and faster cellular uptake than the nanospheres or long nanorods. These results confirmed that tumor selectivity is not only driven by the capacity of filomicelles to evade the immune system, but also by their ability to permeate into different tissues, which is correlated to their shape. Moreover, the influence of nanoparticle shape on uptake and cellular response was also highlighted by Zhang and coworkers [96]. While both spherical and needle-shaped PLGA-PEG nanoparticles entered cells via endocytosis, only needle-shaped nanoparticles were found to induce significant cytotoxicity [96]. This effect seemed to be induced through lysosome disruption, although this mechanism may be strongly dependent on the rigidity of the material used.

## 4. Effect of Particle Shape on Drug Delivery Properties

Due to their advantages in overcoming the different biological barriers, non-spherical polymeric nanoparticles are promising drug nanodelivery systems. Several studies investigated the effect of particle shape on drug delivery properties. Both drug encapsulation and delivery are affected by the geometry of the nanocarriers, together with the capacity of the hydrophobic core to entrap hydrophobic drugs, and the polymer degradation kinetics. [11,12,76]. The main drug delivery properties of non-spherical polymeric nanocarriers are summarized in Table 3. 

Numerous investigations were performed on filomicelles obtained by amphiphilic copolymers self-assembly. The results demonstrated that filomicelles allow higher anticancer drugs encapsulation than spherical equivalents, resulting in a greater apoptotic effect on tumor cells [97]. Compared to spherical micelles, filomicelles present a higher surface-to-volume ratio [59], and, most importantly, a larger size of hydrophobic blocks (i.e. a higher packing parameter) [98], which may favor the drug–copolymer interactions and therefore the final drug partition coefficient [99].

Several studies on PCL-PEG based filomicelles for anticancer drug encapsulation showed that higher drug loading contents (DL, %wt.) and encapsulation efficiency (EE, %wt.) can be achieved compared to spherical counterparts [15,59,61,100,101,102,103]. PTX was one of the most investigated anticancer drugs [15,59,100]. Geng and coworkers performed both in vitro and in vivo studies on PTX-loaded filomicelles, which presented almost two times higher DL than spherical micelles for different initial concentrations [100]. The filomicelle capacity to encapsulate PTX was closely linked to the hydrophobic core diameter, reaching a DL = 4.5% and 6.7% for filomicelles with PCL core diameter of 11nm and 29nm, respectively [59]. This conclusion was confirmed by Sun et al. for PCL-PEG-based filomicelles, since PTX drug loading (DL above 10% and EE higher than 63%) and release were closely related to the length of PCL core and copolymer composition [103]. In vitro and in vivo studies on PTX-loaded PCL-PEG filomicelles demonstrated a reduced PTX toxicity and greater anticancer activity than spherical micelles or free drug [15,61,100]. In the case of PCL-PEG filomicelles loaded with dexamethasone, DL up to 10% and EE over 90% were obtained, which correspond to higher values than those obtained with spherical micelles [102].

A slight change in copolymer composition seems to have an important impact on drug encapsulation in non-spherical nanocarriers [104]. In fact, the amount of drug loaded may increase when the hydrophobicity of the core is enhanced [105]. For instance, elongated micelles with poly(styrene oxide) blocks are able to solubilize four times more than micelles with a poly(butylene oxide) core [106]. Drug loading may also depend on their amorphism; the replacement of the semicrystalline PCL with other amorphous polyesters such as poly(δ-decalactone) increased DL [107].

Nair et al. investigated PTX loading capacity of PEG-PBCL filomicelles, containing an aromatic group in the hydrophobic core, compared to equivalent PEG-PCL filomicelles. The former presented 40% higher EE than the PEG-PCL filomicelles. In vitro tests on human lung cancer cells and tests on mice bearing tumor xenografts highlighted the better efficacy of PEG-PBCL micelles to induce tumor cells apoptosis and tumor shrinkage over time [101].

PLA-PEG-based filomicelles loaded with betulin derivative also reached high DL (around 20%) and EE near 100%, leading to significant apoptosis of carcinoma cells in vitro [108].

Investigations on synergetic multidrug loading on filomicelles were also performed as combination therapy to overcome tumor heterogeneity and tumor resistance. PEG-PCL filomicelles loaded with PTX and retinoic acid [109] and PLA-PEG filomicelles co-loaded with PTX and 17-AAG, or triple combination with rapamycin [110] showed interesting DL (around 10%) and EE (above 68%). These studies demonstrated that drug–drug and drug–polymer interactions play an important role both on drug-cocktail encapsulation and release.

Worm-like polymeric micelles based on poly(2-oxazoline)s (POx) poly(2-methyl-2-oxazoline-block-2-butyl-2-oxazoline-block-2-methyl-2-oxazoline) (P(MeOx-b-BuOx-b-MeOx) were used to achieve co-loading (over 50%wt.) of etoposide (ETO) and an alkylated cisplatin prodrug (C6CP) [111], improving their pharmacokinetics, tumor distribution, and antitumor activity in animal models of small/non-small cell lung cancer. Drug–polymer interaction also affected particle morphology; starting from spheres with inside C6CP, they became worms after addition of ETO [111]. On the contrary, a transition from partially worm-like to spherical morphology was obtained from unloaded polymer upon encapsulation of small amounts of PTX [112].

Chen et al. worked on poly(etheranhydrides) terpolymers spheres, nanorods and filomicelles (with same diameter) loaded with DOX. They obtained the highest encapsulation values with filomicelles (DL = 10.6%, EE = 75.1%), then with nanorods (DL = 7.4%, EE = 68.8%) compared to spherical counterparts (DL = 5.2%, EE = 70%). The antitumor effect on tumor-bearing mice followed the same trend, with the highest decrease in tumor volume for DOX-loaded filomicelles [113]. Li et al. found similar results but with higher DOX encapsulation values for PCL-PEG long nanorods (DL = 8.4%, EE = 92.3%) and short nanorods (DL = 7.3%, EE = 80.2%) compared to nanospheres (DL = 4.5%, EE = 49.5%) [17].

In the case of unimolecular bottlebrush micelles, a relatively high drug loading capacity (up to ca. 25%) was obtained independently of morphology (sphere, rod, and worm) [114]. These nanoparticles were obtained using poly(2-hydroxyethyl methacrylate) (PHEMA) as backbones and poly(tert-butyl acrylate)-block-poly(ethylene glycol) (PtBA-b-PEG), loaded with IR780 photothermal agent. The rod-like shape performed favorable behavior for cellular uptake in 2D culture and spheroid penetration in vitro, preferential tumor accumulation in mice and photothermal therapeutic efficacy in MCF-7 tumor xenograft model in vivo.

A tri-component polymer brush composed of a polybenzofulvene copolymer bearing low molecular weight hyaluronic acid and oligo-PEG fractions was also proposed as a nanocarrier for targeted delivery of DOX (DL ~13%), since this nanomaterial was able to be internalized into cancer cells by CD44 receptor-mediated uptake [115].

Drug loading, together with drug−polymer interaction and drug diffusion coefficient, are key factors that influence the release profile in most nanocarrier systems. Clearly, particle shape is directly related to the surface-to-volume ratio, which makes an important contribution to the drug diffusion mechanisms [83]. Surface-to-volume ratio may also affect the kinetics of nanoparticle degradation and, as a result, drug release. In PEO-b-PCL worm-like micelles, a relatively rapid degradation to spherical micelles was observed, as a result of hydrolytic degradation of PCL block [59,104]. The in vitro release kinetics of PTX from these worm micelles showed an initial burst release due to the weak localization of some of the drug in the core–corona interface region, followed by a much slower and sustained release.

pH transitions are often exploited by nanocarriers to selectively deliver drug in acidic environments, such as in tumors and in endosomes. Therefore, in vitro release tests are often carried out to assess the effect of pH on polymer degradability [98], self-assembly [107], drug solubility [105,116], and therefore on the release profile.

The amount of PTX released by the PLA-PEG filomicelles was less than that released by the spherical micelles at fixed pH. In particular, ~15% release at pH 5.5 and ~22% at pH 3.0 were observed for filomicelles in 71 days, in contrast to 36% at pH 5.5 and 63% at pH 3.0 for spherical micelles, which was in agreement with the faster degradation of spherical micelles [12].

pH-responsive worm-like micelles (diameter of ~20 nm and length 50−200 nm) were obtained from methoxy poly(ethylene glycol)-block-poly(2-diisopropyl methacrylate) (mPEG-b-PDPA) loaded with succinobucol (DL about 15% and EE about 93%), a selective inhibitor of vascular cell adhesion molecule-1 (VCAM-1), which was selected as a potential candidate against lung metastasis of breast cancer [107]. A pH-sensitive drug release was achieved in response to acidic intracellular environments, reducing the expression of the metastasis-associated VCAM-1, thus inhibiting the migration of metastatic 4T1 breast cancer cells. These particles induced a higher specific accumulation in lung, and higher delivery on the lung sites metastases, which allowed a reduction of the brain tumor close to 86%.

Worm-like micelles (diameter ~20 nm and length 60−600 nm) composed of pH-responsive mPEG-b-PDPA copolymer and loaded with cyclic RGD peptide targeted cytotoxic emtansine (DM1) conjugates (RGD-DM1), were developed for brain tumor targeting [117]. The nanoworms dissociated at intracellular acidic environments to release RGD-DM1, which was further degraded into DM1 by disulfide cleavage. These nanocarriers enhanced drug delivery to the brain, with deep penetration into brain tumor mass, and efficient internalization into glioma cells, leading to almost 90% inhibition on tumor progression in an orthotopic brain tumor model.

Photosensitizing drug-carrying worm-like micelles were also obtained using a pH-sensitive miktoarm block copolymer consisting of one methoxy PEG block and two 3-diethylaminopropylated poly(l-lysine) [poly(Lys-DEAP)] blocks [118]. These worm-like micelles disintegrate in the acidic environment of solid tumors, resulting in targeted delivery of the photosensitizing drug, which reduced approximately five times the tumor volume in nude mice.

Finally, Enlow and coworkers investigated PLGA cylinders manufactured by PRINT method and loaded with docetaxel. They studied different initial loading ratio between 0 and 40% and obtained EE higher than 90% for each ratio, indicating that PRINT PLGA cylinders are interesting nanocarriers for high loading capacities [39]. Chu et al. found similar DL (33.5% for short nanorods and 45.2% for longer nanorods) for Docetaxel-loaded PRINT PLGA nanorods but with a lower EE caused by the washing steps performed during the process [38].

**Table 3:** Non-spherical nanocarriers for drug delivery applications.

Fabrication technique	Shape	Material	Drug	Target	Ref.
Conventional self-assembly	Filomicelles	PEG-PEE, PEG-PCL	PTX	human-derived tumors in mice	[15]
	Filomicelles	PEG-b-P(CPTKMA-co-PEMA)	Conjugated CPT	Tumor bearing mice	[16]
	Nanorods	PEG-PCL	DOX	HeLa, HepG2, OB cells; Balb/c mice bearing H22 tumor xenografts.	[17]
	Crosslinked wormlike vesicles	PEG-PLA-PEG	DOX	HeLa cells	[19]
	Filomicelles	PEG-PPS	Chloroquine	plasmacytoid dendritic cells	[22]
	Worm-like/rod-like vesicles	POEGMA-b-P(ST-co-VBA)	DOX	MCF-7 cells	[32]
	Filomicelles	PEG-PCL	PTX	A549 Tumor-bearing mice	[61]
	Tubular polymersomes /worm-like micelles	PEG, PTMC, PCL, and PDLLA block copolymers	DEX	retinal (ARPE-19) cells; ex vivo porcine eyes	[65]
	Nanorods	PEG-xCPT	CPT/DOX	MCF-7/ADR cancer cells	[75]
	Filomicelles	PEG-PCL, PEG-PBCL	PTX	A549 lung cancer cells, EC4 liver cancer cells	[100,101]
	Filomicelles	PEG-PLA	Betulin derivative	HeLa cells	[108]
	Filomicelles	PEG-PCL	PTX, retinoic acid	A549, HepG2, U2os, EC4	[109]
	Filomicelles	PEG-PLGA	PTX, 17AAG, rapamycin	CaCo-2 human colorectal adenocarcinoma cells	[110]
	Filomicelles	P(MeOx-b-BuOx-b-MeOx)	ETO, C6CP, PTX	Small/ non-small cell lung cancer models	[111]
	Filomicelles, nanorods	poly(ether-anhydrides)	DOX	Murine breast cancer model	[113]
	pH-responsive wormlike micelles	PEG-PDPA	RGD-DM1	Orthotopic brain tumor model	[117]
	pH-responsive wormlike micelles	mPEG-ser-[poly(Lys-DEAP)]_2_	Chlorin e6	KB cells and tumor-bearing mice	[118]
	pH-responsive wormlike micelles	PEG-PDPA	Succinobucol	Metastatic breast cancer Model	[119]
Unimolecular polymer brushes	Nanorods	PNB-g-PGA	Conjugated CPT	HeLa, LS174T, and HEK cells	[31]
	Nanoworms, lamellae, vesicles	PHPMA-b-(NBMA-*co*-CMA)	DOX	HeLa cells	[33]
	Cylindrical bottlebrushes	cellulose-g-(CPT-b-OEGMA)	Conjugated CPT	MCF-7 induced multicellular spheroids and tumor-bearing mice	[73]
	pH sensitive nanorods	PHF-g-(PCL-PEG)	DOX	A459 human lung cancer cells	[105]
	Nanorods, Nanoworms	PHEMA-g-(PtBA-b-PEG)	IR780	photothermal therapy in MCF-7 tumor models	[114]
	Cylindrical brushes	HA-polybenzofulvene	DOX	HCT116, MCF-7, 16HBE cell lines	[115]
PRINT	Nanorods	PLGA	Docetaxel	Human ovarian carcinoma cells; Mice bearing tumor xenografts	[38,39]

## 5. Conclusions

Several studies have shown that the presented manufacturing methods allow the design of anisotropic polymeric nanocarriers with precise shape and size, obtaining mainly nanoworms, nanorods and nanodisks. These methods can be applied to different types of biocompatible polymers and studies are still ongoing to expand the polymer library. Different works has shown that nanocarrier shape is a critical parameter, along with size, to tailor their biological interactions and their drug delivery system properties. Indeed, the shape affects the nanoparticle clearance by biological systems, their transport to the targeted place and their biodistribution and targeting capacity. Short filomicelles, nanorods and nanodisks possess interesting properties which may be exploited to deliver therapeutics efficiently at the tumor site and with reduced side effects. Moreover, anisotropic nanocarriers generally present higher drug loading and encapsulation efficiency compared to spherical nanoparticles. In vitro and in vivo studies also showed higher targeting efficiency on tumor cells and tissues. This review highlights the importance of the shape in the design of polymeric nanocarriers for drug delivery systems. However, nanocarriers are complex materials and it is difficult to isolate the shape effect from their size, surface chemistry, density, and rigidity. Despite the clear involvement of particle shape in biological processes, very few examples of non-spherical nanoparticles have entered the clinical stage. Further research in this field is therefore required to speed up the translation of non-spherical polymer nanoparticles into the clinic.

## Figures and Tables

**Table 1:** Manufacturing methods for non-spherical nanoparticles of different shape, size and material.

Fabrication Technique	Non-spherical Shapes	Size Range	Materials	Ref.
**Self-assembly**				
Conventional	Filomicelles/worms Short and Long rods Vesicles	Ø = 20–60 nm L = 100–1800 nm	PS-PAA, PS-PEO, PEE-PEG, PCL-PEG, PCL-PEO, PEG-PPS, PEG-PLA PAA-PMA-PS, PEG-PLA-PEG	[14,15,16,17,18,19,20,21,22,23,24]
Nucleic acid complexation	Nanorods, nanoworms	Ø < 80 nm L > 140 nm	DNA/PEG-PPA, DNA/(lPEI)-PEG	[25,26]
Unimolecular–polymer brushes	Worms Cylindrical	Ø = 17–35 nm L = 35–1200 nm	PCL-(PEGMA-*co*-GMA), PNB-g-(PS-b-PMA-b-PAA), PGMA-g-PEG, PNB-g-PGA	[27,28,29,30,31]
**PISA**	Worms Rods Vesicles	Ø = 20–32 nm L = 90–635 nm	POEGMA-P(ST-*co*-VBA), PHPMA-(NBMA-*co*-CMA), PMeOx-b-PiPrOx	[32,33,34]
**Membrane stretching**	Disks Rods	Ø = 100–240 nm L = 360–500 nm	PS, PLGA	[35,36,37]
**PRINT**	Trapezoid, cones Rods Cylinders	80–600 nm	PEG, PLA, PLGA	[38,39,40,41]

**Table 2:** Effect of nanocarriers shape on biological processes compare to sphere counterparts.

Non-Spherical Nanocarriers	Effect on Biological Processes	Ref.
Long Filomicelles (>10 µm length)	⋅ Prolonged circulation time ⋅ Reduced circulation time (for polymer brushes) ⋅ High phagocytosis rate ⋅ Reduced target selectivity and tumor internalization ⋅ High accumulation in liver and spleen	[15,16,17,28,29,30,56,57,58,59,60,61,62,63,64,65,66,67]
Short Filomicelles, Nanorods Ellipsoids (<10 µm length)	⋅ Prolonged circulation time ⋅ Greater vascular margination ⋅ Reduced phagocytosis rate ⋅ High tumor cells internalization ⋅ Low accumulation in liver and spleen ⋅ High retention time in intestinal cells	[16,17,28,29,30,35,36,56,68,69,70,71,72,73,74,75,76,77]
Nanodisks	⋅ Prolonged circulation time ⋅ Greater vascular margination ⋅ Reduced phagocytosis rate ⋅ High tumor cells internalization ⋅ Lower specific uptake than nanorods	[35,56,68,69,71,78,79,80,81,82]

## Data Availability

Not applicable.
